# Microplastic contamination of lettuces grown in urban vegetable gardens in Lisbon (Portugal)

**DOI:** 10.1038/s41598-023-40840-z

**Published:** 2023-08-31

**Authors:** Nuno Canha, Mehriban Jafarova, Lisa Grifoni, Carla A. Gamelas, Luís C. Alves, Susana Marta Almeida, Stefano Loppi

**Affiliations:** 1 Centro de Ciências e Tecnologias Nucleares C2TN, Instituto Superior Técnico, Universidade de Lisboa, Estrada Nacional 10, Km 139.7, 2695-066 Bobadela LRS, Portugal; 2 University of Siena, Siena, Italy; 3 Istituto Nazionale Geofisica e Vulcanologia (INGV), 605, Via di Vigna Murata, 00143 Roma, Italy; 4 Instituto Politécnico de Setúbal, Escola Superior de Tecnologia de Setúbal, Centro de Investigação em Energia e Ambiente, IPS Campus, 2914-508 Setúbal, Portugal

**Keywords:** Environmental sciences, Environmental chemistry, Environmental impact

## Abstract

Urban vegetable gardens are very often a feature of cities that want to offer their citizens a more sustainable lifestyle by producing their own food products. However, cities can have significant pollution levels (or pollution hotspots) due to specific sources of pollution, such as traffic. Among the various pollutants, microplastics (MPs) are emerging as a consensual concern due to the awareness of the environmental contamination, their bioaccumulation potential and human intake, and, consequently unknown human health impacts. The present study compared the content of MPs in lettuce plants cultivated in Lisbon urban gardens with those cultivated in a rural area, as well as samples bought in supermarkets. Microplastics were detected in all washed leaves, with mean levels ranging from 6.3 ± 6.2 to 29.4 ± 18.2 MPs/g. Lettuce grown in urban gardens from areas with high traffic density showed higher MPs levels. Weak positive Spearman’s rank correlations were found between MPs content and concentrations of Cu and S (determined by Particle Induced X-Ray Emission, PIXE), suggesting a possible role of traffic contribution to MPs levels, as both elements are considered traffic-source tracers. These results contribute to shed light on the MP contamination of vegetables grown in such urban environments, that may represent a potential MP exposure route through the dietary intake, corresponding to a 70% increase in annual MP intake compared to lettuces bought in supermarkets.

## Main

Urban vegetable gardens are seen as a sustainable strategy for people living in large cities to grow their own food^1^, create green spaces and also promote social connections between residents and a community feeling^2^. Moreover, growing food in urban gardens is also considered to provide health and wellbeing benefits to citizens, as well as promoting environmental sustainability^3^.

Microplastics (MPs, plastic pieces < 5 mm) are a growing health concern due to their contamination of the environment, from water systems to soil and air^4^. One of the potential sources of MPs in the air is traffic^5^ (e.g. tire wear), and considering that urban vegetable gardens are sometimes located in city areas with considerable traffic levels, the hypothesis of bio-accumulation of MPs by the vegetables grown in such settings should be considered and evaluated to understand consumer exposure to MPs and their potential intake via the food chain^6^.

A study carried out in Italy, where edible fruit and vegetables from local markets were analysed for contamination by micro (M) and nano (N) plastics (with a size lower than 10 μm) using the SEM–EDX method, found that apples were the most contaminated samples (195,500 ± 128,687 MP/NPs per gram), while lettuces were the least contaminated (50,550 ± 25,011 MP/NPs per gram)^7^. It is important to highlight that the levels found are somehow high, when compared with other studies in food products^8, 9^, such as levels between 0 and 20 MPs/g in food products as salt, fish sauce, salted seafood, seaweed and honey^10^. However, this study included both MPs and NPs and used a new analytical method^11^, which may have contributed to these results^12^. Therefore, the use of different analytical techniques for the assessment of MP/NPs may also lead to variations in the concentration and characteristics of the detected MP/NPs^9^, highlighting the need to develop standardised methodologies for MP/NP research in this type of samples^13^.

It is known that MPs can accumulate to a large extent in lettuce leaves, mainly by stomatal uptake and cuticle entry^14^. This highlights the potential for follicular uptake of MP deposition on the leaf surface.

Therefore, in order to understand the potential exposure of citizens to MPs through dietary intake of lettuce grown in urban vegetable gardens, it is essential to characterise the level of MPs accumulated by such products. This was the main goal of the present study, which allows to provide insights of the exposure to MPs by this route and contributes to assess the real human exposure to MPs (considering the different intake routes).

## Results

A total of 101 MP particles were detected in the 14 different lettuce samples, of which 90.2% were classified as fibers and 9.8% as fragments. Of the 42 sub-samples analyzed (considering both types of lettuces analysed, namely, smooth leaf lettuce—SLL—and beaded leaf lettuce—BLL), 6 were completely free of fibers and fragments (only happened in one sub-sample of samples U2-SLL and U5-SLL and of samples of both lettuce types of U7 and C).

**Figure 1:**
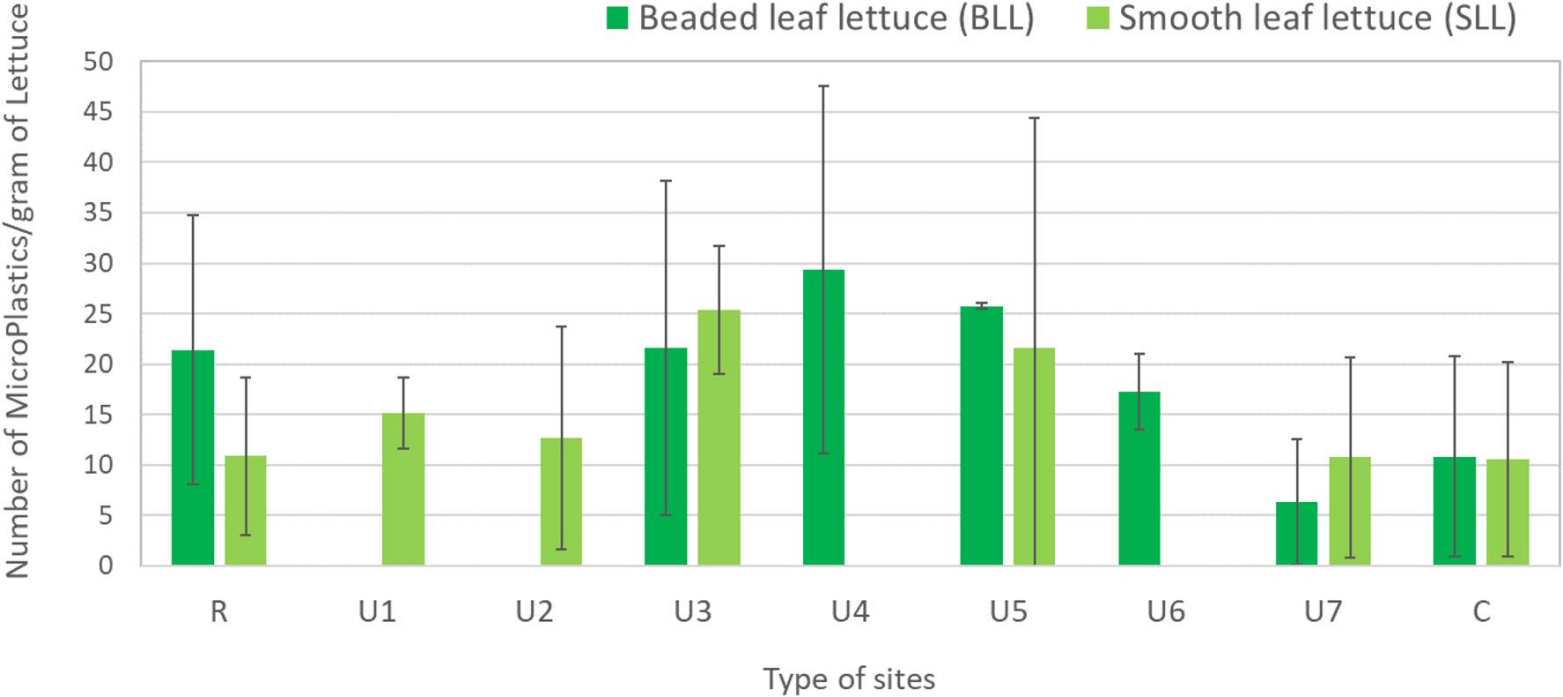
MPs per gram of dry mass (mean ± standard deviation) in two different types of lettuce (BLL and SLL) grown in the different environments (R—Rural, U1…U7—Urban, C—Supermarket).

Overall, MP levels were not significantly different between lettuce types, with BLL presenting a mean contamination of 18.9 ± 8.1 MPs/g, and SLL 15.3 ± 5.9 MPs/g (Fig. 1).

Commercial samples presented similar levels of MPs per gram for both types of lettuce (10.8 ± 9.9 MPs/g and 10.6 ± 9.7 MPs/g for BLL and SLL, respectively). Samples grown in the rural area showed 10.9 ± 7.8 MPs/g for SLL (similar to commercial samples), but BLL presented almost double levels (21.4 ± 13.3 MPs/g).

Lettuce grown in urban vegetable gardens typically showed higher MP contamination, except for site U7, where the SLL levels were similar to the commercial levels, namely 10.8 ± 9.9 MPs/g, and BLL presented the lower level found in this study for all samples: 6.3 ± 6.2 MPs/g. The remaining urban sites presented levels between 12.7 MPs/g (U2) and 29.4 (U4) MPs/g. Levels above 20 MPs/g, for both lettuce types, were found at 3 sites (U3, U4 and U5) that are close to busy roads.

To disclose possible sources of MPs contaminating our lettuce samples, their elemental content was also measured (Table 1), and Spearman's Rank correlation coefficients between MPs and each chemical element were determined. Weak positive associations, but not statistically significant, were found between MPs levels and Cu (0.25) and S (0.31).

**Table 1:** Mean levels of chemical elements and MPs in the studied samples, along with Spearman’s rank between them (correlation coefficients and their *p*-value).

Parameter/Element	Unit	Mean ± SD (Min—Max)	Spearman's Rank between MPs and chemical elements	
			Correlation coefficient	*p*-value
Microplastics (MPs)	MPs/g	19.7 ± 6.7 (9.5–32.4)	1.00	–
Al	µg/g	333 ± 158 (79–604)	− 0.15	0.633
Br	µg/g	15.3 ± 10.5 (4.5–42.6)	− 0.27	0.342
Ca	mg/g	14.0 ± 3.5 (7.6–20.1)	− 0.05	0.864
Cl	mg/g	26.6 ± 5.4 (20.0–38.5)	− 0.23	0.427
Cu	µg/g	13.7 ± 20.1 (5.0–82.7)	0.25	0.383
Fe	µg/g	241 ± 159 (69–699)	− 0.42	0.135
K	mg/g	60.7 ± 12.2 (31.5–72.9)	− 0.11	0.703
Mg	mg/g	1.40 ± 0.37 (0.86–1.99)	− 0.28	0.334
Ni	µg/g	2.19 ± 2.67 (0.77–11.07)	− 0.15	0.615
P	mg/g	3.38 ± 0.78 (2.14–5.11)	− 0.23	0.427
Rb	µg/g	39.9 ± 43.3 (10.0–146.2)	− 0.18	0.543
S	mg/g	3.32 ± 1.10 (1.78–5.56)	0.31	0.288
Si	mg/g	1.18 ± 0.83 (0.38–3.54)	− 0.14	0.626
Sr	µg/g	55.4 ± 29.7 (15.8–113.3)	− 0.26	0.375
Ti	µg/g	30.3 ± 36.4 (2.6–141.0)	− 0.41	0.191
Zn	µg/g	54.1 ± 26.6 (10.1–98.0)	0.02	0.958

## Discussion

MPs found in lettuce grown in the urban area showed a wide range of concentrations, with differences between locations probably due to proximity to roads with high traffic levels, combined with low atmospheric dispersion conditions. For instance, U7 is located close to a highway, but probably due to the open conditions, the lettuces collected in this urban garden presented the lowest levels of MPs. The highest levels were found at sites U3-U5, located nearby high traffic roads, or next to commercial and residential buildings. The urban garden U5 is located in a square surrounded by residential buildings and at 300 m from the main highway that crosses the city of Lisbon (Segunda Circular).

The levels found in lettuce samples in the present study agree with the levels found in lichens *Evernia prunastri* that were exposed during three months in urban parks in Milan (Italy)^15^, at a height of 1.5–2 m above the ground, where 26 ± 1 MPs/g were found. However, lichens exposed in other areas of this city showed higher MP levels, namely, 44 ± 1 MPs/g in the city centre and 56 ± 5 MPs/g in the periphery. It is important to highlight that the potential mechanisms of MPs uptake between lichens and lettuces are not exactly the same, but have similarities. For both lichens and lettuces, the uptake mechanism is by foliar uptake (MPs accumulated after deposition on the leaf surface). However, lettuce has an additional mechanism, namely, by soil-root transfer (in this way, MPs that have fall out in the soil can enter in the plant)^16^. Despite this difference, both species can accumulate MP by the described processes. Another difference is, naturally, the height where the lettuces and lichens are located. Lettuces are grown at the soil level while lichens were deployed at a higher height (1.5–2 m) from the soil (tied to the branches of trees). However, despite the differences between lettuces and lichens, the comparison may be helpful to identify the typical range of MPs found in specific environments.

Spearman’s correlations showed a weak positive association between the levels of MPs and Cu (0.25) and S (0.31). Although not statistically significant, these two elements may provide some indications about the sources of MPs in lettuce, as both elements are typically associated with traffic levels. Sulphur is emitted by diesel automobiles and Cu is associated with mechanical abrasion of brakes^17^. This hypothesis should be explored in the future by increasing the number of samples (which is a limitation of the present study), thus providing insights into the contribution of traffic to the levels of MPs in nearby urban vegetable gardens.

In fact, tire wear is considered to be one of the largest sources of microplastics entering into the environment, due to the release of tire wear particles (TWP) formed by the mechanical abrasion of car tires on the road surface^5^, whereas Cu is considered a source-tracer^17^. It is known that TWP-derived compounds (which can be deposited in soils) can be taken up by edible plants (such as lettuce) and their metabolization products can accumulate in their leaves^18^. A study conducted in China (suburb Wuhan) found that soils of vegetable farmlands had MPs levels in the range of 0.32–12.56 MPs/g, with a higher abundance of MPs nearby suburban roads (2.45 MPs/g) when compared with residential areas (1.37 MPs/g)^19^.

It is also important to highlight that none of the studied vegetable gardens used mulching, suggesting that potential contamination of soils by MPs should be from sources other than mulching. In fact, several studies have identified mulching as a major source of MPs in soils, where plastic film-derived MPs can migrate down the soil profile^20^ and may affect crop productivity by influencing soil physicochemical properties^21^.The results of the present study showed that the mean MPs contamination of lettuce samples from rural (16.2 ± 7.5 MPs/g) and urban (18.6 ± 7.4 MPs/g) environments were similar and both were higher than those from commercial settings (10.7 ± 0.2 MPs/g). It would be expected that MPs contamination levels would be similar between rural and commercial environments, but this was not observed in the present study. Typically, commercial lettuce is grown in agricultural and open areas with low levels of nearby sources of air pollution (e.g., no traffic). A possible explanation for the higher levels in the rural setting (which was a typical small village in inland Portugal) may be the local sources of air pollution. Specifically, the rural samples were collected in a vegetable garden of a house that was located in the middle of the village (with 811 inhabitants)^22^, surrounded by other houses. This means that although traffic is low, its influence may not be discarded. In addition, other types of local sources may affect air quality in these rural areas, such as open burning of vegetable debris and solid household waste (including plastic products) in the backyards of houses, which is a common practice in rural communities^23^. Bottom ash from incineration of plastic waste is known to be a potential source of microplastics released into the environment^24^, which may explain the higher levels of MPs contamination in the rural environment than it was expected.

Considering that European citizens consume 22 g of lettuce per day^25^ and that lettuce contains around 95% of water ^26^, an adult consumes approximately 402 g of lettuce (dry weight) per year. This means that, considering that lettuces are acquired in commercial settings, where the mean MPs content found in the present study was 10.7 ± 0.2 MPs/g, an adult ingests 4,300 ± 79 MP particles per year through dietary intake. However, considering lettuces cultivated in both rural and urban vegetable gardens (with MP levels of 16.2 ± 7.5 MPs/g and 18.6 ± 7.4 MPs/g for rural and urban environments, respectively, and with an overall mean MP levels of 18.2 ± 7.1 MPs/g for both settings), MP ingestion increases by 70%, with an annual ingestion of 7299 ± 2859 MP particles per year due to dietary intake, based on lettuce consumption alone.

Another relevant issue is the preparation of lettuce for human consumption. The washing process may affect the levels of MPs in lettuce leaves. The success of different washing procedures of lettuce to reduce MPs (that were previously sprayed to their surface) was already studied^27^. Of the three different washing methods studied (water rinsing, ultrasonic vibration cleaning, and edible detergent cleaning), it was found that simple rinsing gave the lowest removal efficiency of MP particles from lettuce leaves, followed by ultrasonic vibratory cleaning (4 times better than only being washed) and, then, detergent washing provided the best results (with an increase of 6.9 times than water rinsing). Smaller MP particles (100 nm) were more difficult to clean than the larger ones (500 nm). The increase in efficiency of ultrasonic vibration cleaning is due to the fact that ultrasonic vibrations can partially break the chemical bonds between MPs and lettuce surfaces, while the use of detergents promotes a higher hydrophilicity of the MPs^27^.

Finally, it is important to highlight that the present study should be considered as an exploratory study on the contamination of products from vegetable gardens with MPs, with the major limitation being the limited number of samples. Therefore, further research should be carried out on this topic, with a larger number of samples from different types of environments (rural, urban and commercial), in order to obtain representative results. In any case, despite this limitation, the present work provides, for the first time, valuable insights into the potential MPs contamination of lettuce grown in vegetable gardens.

## Conclusions

This study assessed for the first time the levels of microplastics in lettuce samples grown in urban vegetable gardens, a common practice in cities nowadays. When compared with commercial lettuce from the supermarket, plants grown by citizens in vegetable gardens had more 70% of MPs. Lettuce from vegetable gardens of rural and urban areas presented a similar MP contamination. In the urban setting, lettuces cultivated in areas with high traffic intensity presented higher levels of MPs. Chemical elements associated with traffic, Cu and S, presented some positive associations with MP levels, which may indicate that traffic is a potential local source of MPs. Considering that lettuce is a common vegetable in the human diet, the potential intake of MPs via this route should not be neglected when assessing the overall human exposure to MPs.

## Method

### Type of samples

**Figure 2:**
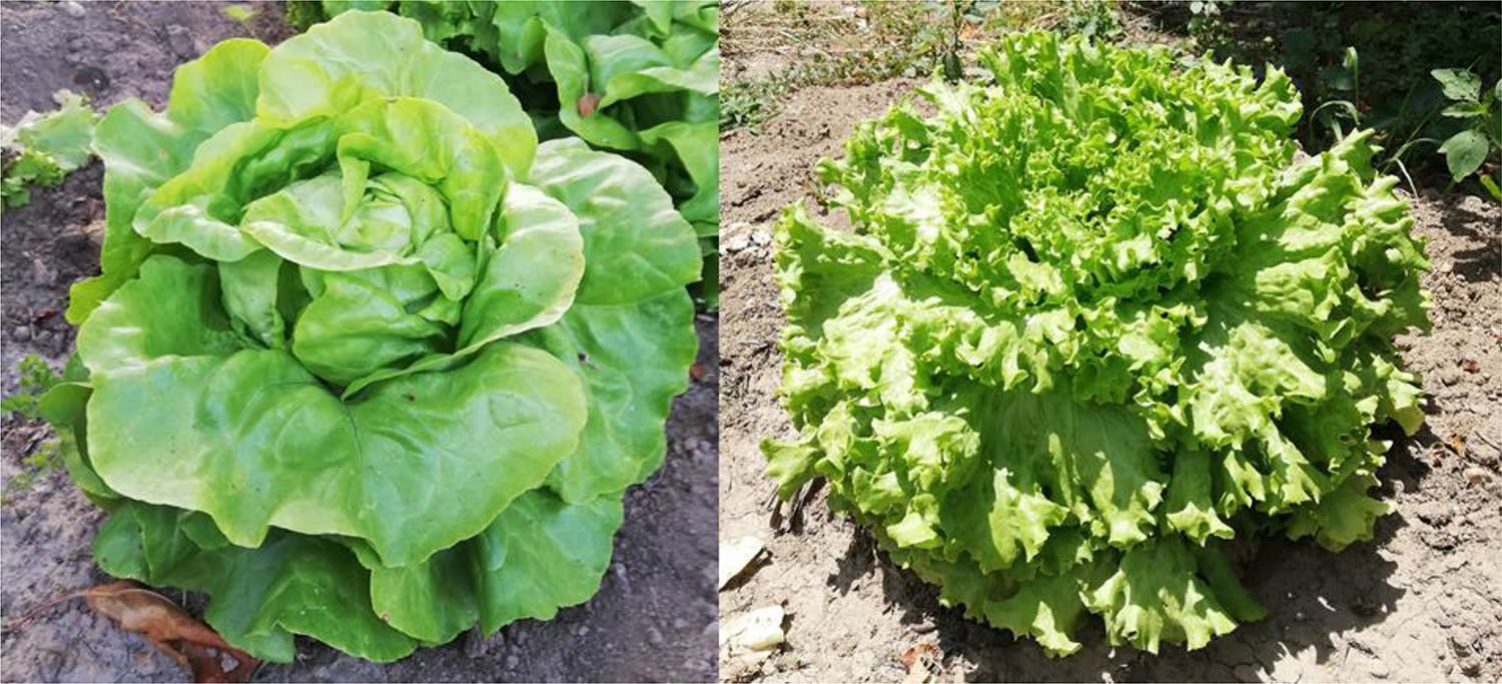
The two cultivars of studied lettuce: (left) Smooth leaf lettuce (SLL) and (right) Beaded leaf lettuce (BLL).

Our study focused on two types of lettuce that are commonly cultivated in urban vegetable gardens in Lisbon, namely, beaded leaf lettuce and smooth leaf lettuce (Fig. 2), two cultivars of *Lactuca sativa L.* Whenever possible, the two types of lettuce were sampled at each location.

### Sampling location

**Figure 3:**
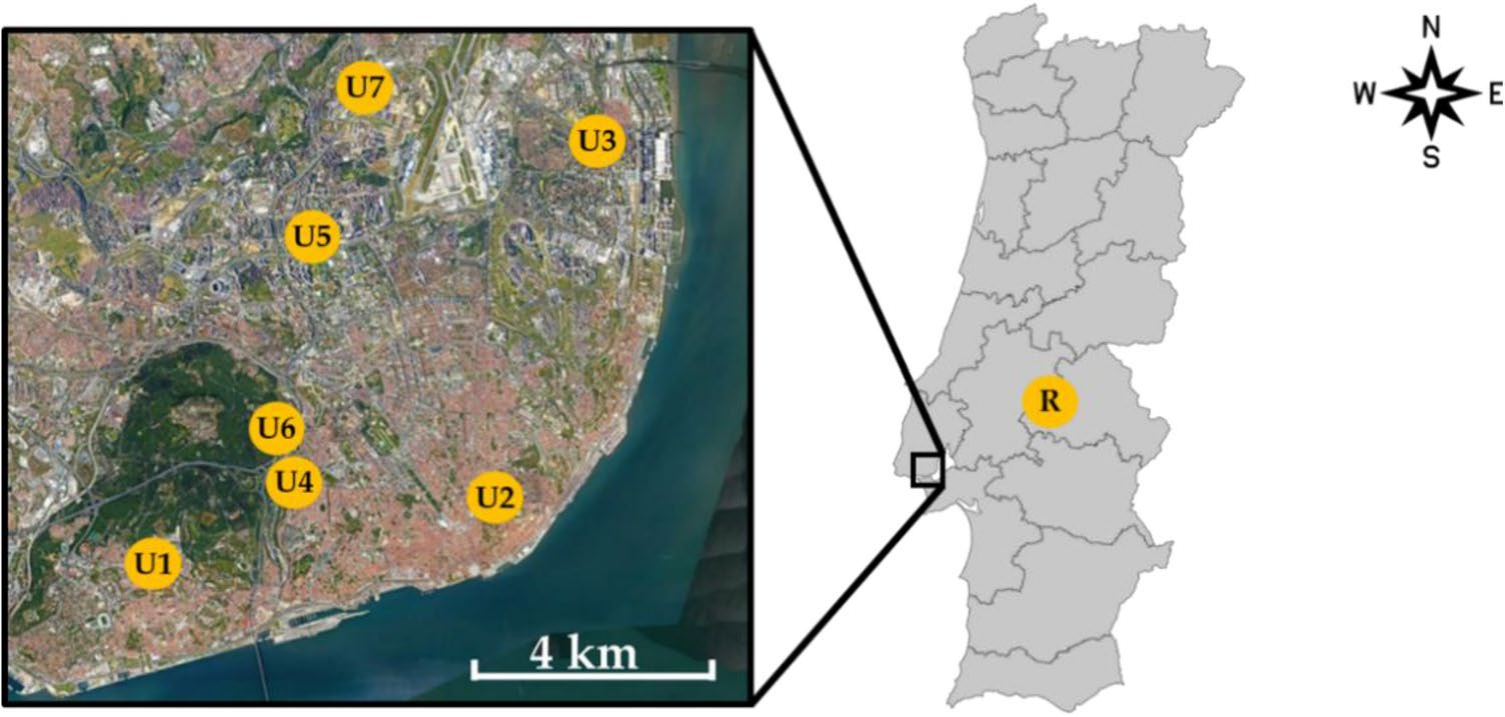
(left) Location of the urban gardens in Lisbon city (from U1 to U7) and (right) location of the rural vegetable garden (map created using Google Earth Pro, version 7.3.6.9345, www.google.com/intl/pt-PT/earth/versions/#earth-pro).

**Figure 4:**
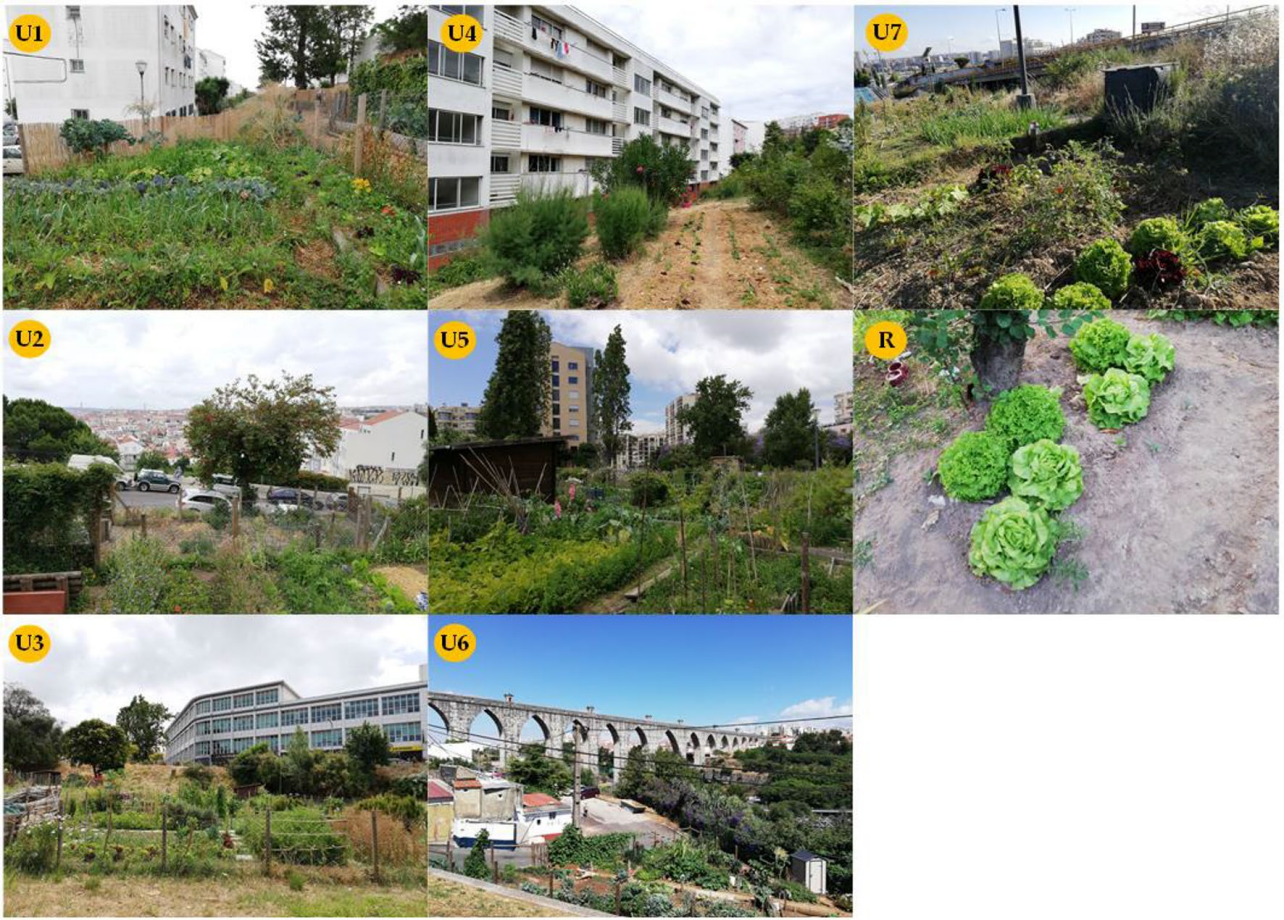
Urban gardens in Lisbon city (from U1 to U7) and rural vegetable garden (R) where lettuces were collected.

Lettuce samples were collected in urban vegetable gardens in the Lisbon area (Portugal), in one rural area of inland Portugal (parish of Foros de Arrão, municipality of Ponte de Sor) and both types of lettuce were also purchased from a major Portuguese supermarket chain supplier. Figure 3 provides the urban and rural locations where lettuce samples were collected and Fig. 4 presents each vegetable garden. No mulching was used in any of the studied vegetable gardens.

### Sample collection and preparation

Sampling of lettuces was carried out in rural and urban areas on 11 and 15 June 2021, respectively. At the field, samples were collected using powderless nitrile gloves, stored in polyethylene bags (not sealed) and kept in a refrigerator. In the following day at the laboratory, lettuce samples were sorted by inner and outer leaves and washed with distilled water. Older and outer leaves that had been in contact with the polyethylene bags during transport were discarded. Afterwards, selected outer leaves samples were freeze-dried and ground into powder in a ball mill with Teflon™ capsules. The grain size of the powder was between 36 and 94 µm. Lettuce of the two types were also purchased from a major Portuguese supermarket chain supplier to be considered as controls, since its cultivation is typically in farming areas and it is a common product purchased by citizens for their food. In total, 14 different samples were considered, as described in Table 2.

**Table 2:** Samples in each site (with its ID) and the type of lettuce collected.

Type of environment		Type of lettuce	
		Beaded leaf	Smooth leaf
Rural	R	✓	✓
Urban	U1		✓
	U2		✓
	U3	✓	✓
	U4	✓	
	U5	✓	✓
	U6	✓	
	U7	✓	✓
Commercial	C	✓	✓

Owners of the vegetable gardens provided voluntarily their consent for the collection of lettuces that they cultivated in their vegetable gardens. In addition, experimental research and field studies on lettuce, including the collection of plant material, complied with institutional, national, and international guidelines and legislation.

### Sample characterisation

#### Microplastic analysis

In the laboratory, powder samples were digested individually using a wet peroxide oxidation method^28, 29^. The samples were then vacuum filtered onto cellulose filter papers (Whatman Grade 1, 1001–090, 11 µm), dyed with 2 mL of Rose Bengal (4,5,6,7-tetrachloro-2′,4′,5′,7′-tetraiodofluorescein, 200 mg/L) to help visual differentiation of synthetic material from organic matter, and the filters placed in glass Petri dishes for storage. The filter papers were examined for MPs under a stereomicroscope (Eurotek OXTL101TUSB equipped with an MDCE-5C digital camera) using a five-criteria method^28, 29^, as briefly described below. Microfibres and fragments that met at least two of the criteria, and were not stained by Rose Bengal, were considered anthropogenic and photographed, and further verified using a hot needle test ^28, 30^. All plastic microfibres and fragments were measured using the open-source image processing software ImageJ. Overall, 42 lettuce samples (14 individual samples with triplicate samples) were analyzed individually. Fibers in the samples had a length in the range between 336 and 2078 µm. Table S1 (in Supplementary Material) presents the results of each type of lettuce sample.

#### Five-criteria method for MPs identification

The five criteria used to identify MPs were:

(i) The fibre is unnaturally coloured (blue, red, green, purple, black, grey) compared with other particles/detritus;
(ii) The fibre appears homogenous in material and texture with no visible cell structure or offshoots and is a consistent width throughout its entire length;
(iii) The fibre remains intact and is not brittle when compressed, tugged or poked with fine tweezers;
(iv) The fibre has a shiny or glossy appearance; and
(v) There is limited fraying with no similarities to natural fibres.

If, at least two of the five criteria are met, then a microfibre or fragment can be classified as a microplastic, after not being stained by Rose Bengal.

#### PIXE analysis

Pellets of 250–300 mg of each sample were prepared for elemental characterisation by proton induced X-ray emission (PIXE) technique, and analysed at the broad beam PIXE line (collimated beam with 5 mm diameter) of the 2.5 MV Van de Graaff accelerator installed at CTN/IST (Portugal). Each pellet was irradiated in vacuum with two different proton beam energies (900 keV and 2 MeV) accordingly with a standard procedure for the analysis of organic samples that was previously used and fully described elsewhere^31, 32^. A total of 16 chemical elements were detected and quantified, namely, Al, Br, Ca, Cl, Cu, Fe, K, Mn, Ni, P, Rb, S, Si, Sr, Ti and Zn. Three replicates were analyzed per type of sample and average and standard deviation results were obtained.

## Data Availability

All data generated or analyzed during this study are included in this published article (and its Supplementary Information files).

## Supplementary Table

Supplementary Information 1.
