# Supplementary material for: Microplastic contamination of lettuces grown in urban vegetable gardens in Lisbon (Portugal)
Source: Sci Rep. 2023 Aug 31;13:14278. doi: 10.1038/s41598-023-40840-z (PMC10471761; doi:10.1038/s41598-023-40840-z)
Supplement: Supplementary file 1 — Supplementary Information 1. [file 41598_2023_40840_MOESM1_ESM.docx]

**Supplementary Material**

Table S1. Microplastics contamination (number of microplastics per gram of dry weight) in lettuce leaves for both of type of lettuces sampled.

|  | **MPs/g** | | | |
| --- | --- | --- | --- | --- |
|  | **Beaded leaf lettuce** | | **Smooth leaf lettuce** | |
| Type of environment | Mean | SD | Mean | SD |
| R | 21.4 | 13.3 | 10.9 | 7.8 |
| U1 | - | - | 15.1 | 3.5 |
| U2 | - | - | 12.7 | 11.0 |
| U3 | 21.6 | 16.6 | 25.4 | 6.4 |
| U4 | 29.4 | 18.2 | - | - |
| U5 | 25.7 | 0.3 | 21.6 | 22.8 |
| U6 | 17.2 | 3.8 | - | - |
| U7 | 6.3 | 6.2 | 10.8 | 9.9 |
| C | 10.8 | 9.9 | 10.6 | 9.7 |
